# Supplementary material for: The interactome of intact mitochondria by cross-linking mass spectrometry provides evidence for coexisting respiratory supercomplexes
Source: Mol Cell Proteomics. 2017 Dec 8;17(2):216–32. doi: 10.1074/mcp.RA117.000470 (PMC5795388; doi:10.1074/mcp.RA117.000470)
Supplement: Supplemental Data [file supp_RA117.000470_133922_0_supp_23978_fzffwf.zip › spectra_annotation/mito_DR_spectra_annotation/310-1-11-1-4-1.pdf]

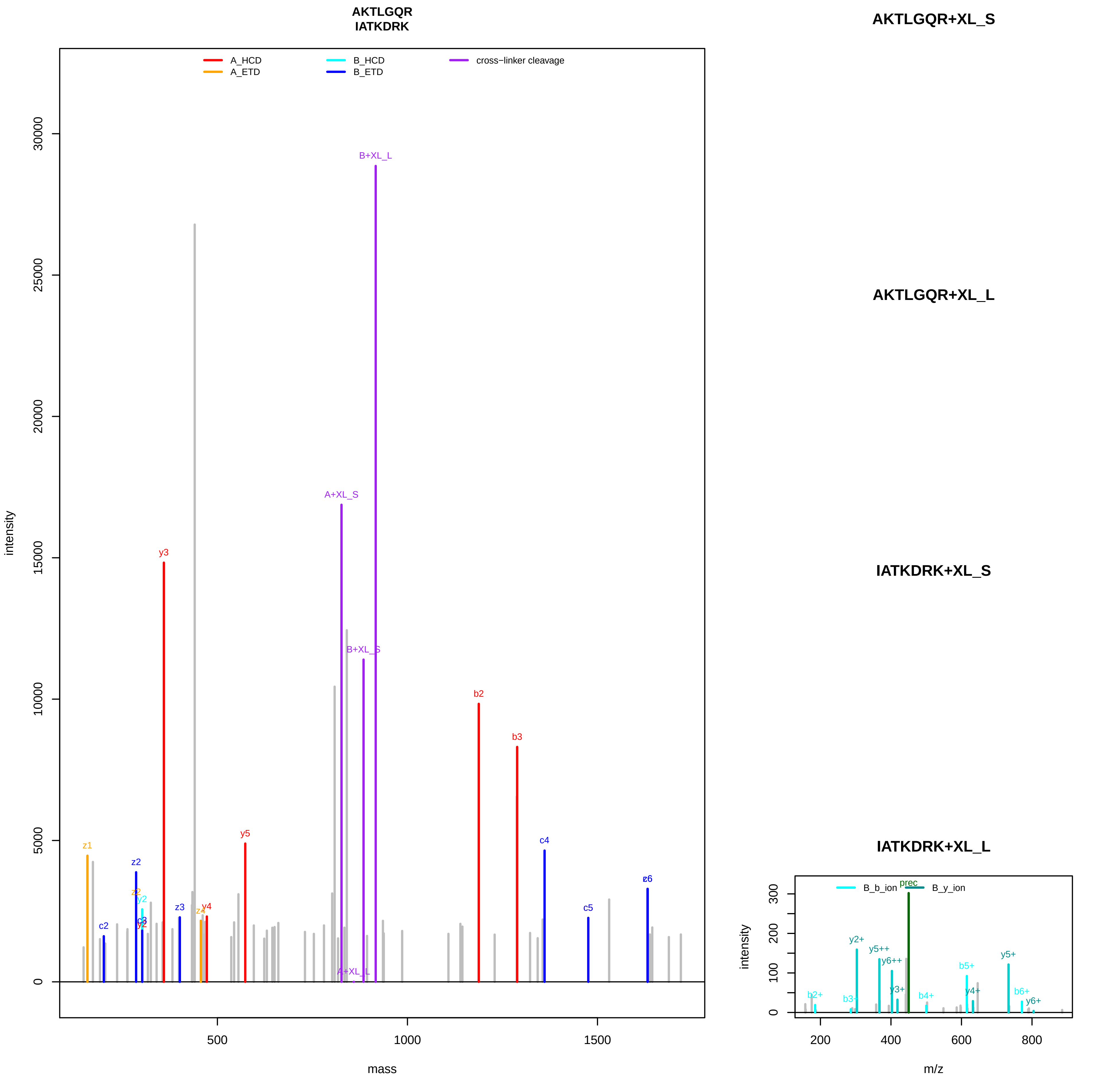
AKTLGQR
IATKDRK
A_HCD
A_ETD
B_HCD
B_ETD
cross−linker cleavage
B+XL_L
A+XL_S
B+XL_S
A+XL_L
y3
y5
y4
b2
b3
z1
z2
z3
z4
c2
c3
c4
c5
c6
z6
y2
intensity
mass
0
5000
10000
15000
20000
25000
30000
500
1000
1500
AKTLGQR+XL_S
AKTLGQR+XL_L
IATKDRK+XL_S
IATKDRK+XL_L
B_b_ion
B_y_ion
prec
y2+
y5++
y6++
y3+
b2+
b3+
b4+
b5+
y4+
y5+
b6+
y6+
intensity
m/z
0
100
200
300
200
400
600
800
